# Immune effector cell associated neurotoxicity syndrome in chimeric antigen receptor-T cell therapy

**DOI:** 10.3389/fimmu.2022.879608

**Published:** 2022-08-23

**Authors:** Robert C. Sterner, Rosalie M. Sterner

**Affiliations:** ^1^ School of Medicine and Public Health, University of Wisconsin-Madison, Madison, WI, United States; ^2^ Department of Surgery, Mayo Clinic, Rochester, MN, United States

**Keywords:** chimeric antigen receptor-T cell (CAR-T cell) therapy, ICANS, immune effector cell associated neurotoxicity syndrome (ICANS), CAR-T cell, neurotoxicity, cytokine release syndrome (CRS)

## Abstract

Chimeric antigen receptor (CAR)-T cell therapy is an emerging staple in the treatment of certain hematological malignancies. While CAR-T cells have produced robust responses in certain hematological malignancies, toxicities associated with the therapy have limited their use. Immune Effector Cell Associated Neurotoxicity Syndrome (ICANS) is a potentially life-threatening neurotoxicity that commonly occurs with CAR-T cell therapy. Here we will discuss ICANS, its treatment, possible mechanisms, and potential solutions to this critical limitation of CAR-T cell therapy. As the field of CAR-T cell therapy evolves, improved treatments and methods to circumvent or overcome ICANS are necessary to improve morbidity, mortality, and decrease the cost of CAR-T cell therapy. This serious, life-threatening side effect needs to be studied to better understand its mechanisms and develop treatments and alternative strategies.

## Introduction

Chimeric antigen receptor (CAR)-T cell therapy is a critical staple therapy for certain hematological malignancies (1–6). T cells are the body’s natural defense against abnormal cells including cancer cells. Typically, this requires the T cell receptor to recognize the peptide antigen of interest in the context of the appropriate major histocompatibility complex. CARs are synthetically engineered receptors expressed in T cells that allow the T cells to recognize independent antigen on the surface of cells. Thus, CAR T cells can recognize antigen and become activated independent of MHC, resulting in robust activation and tumor destruction (7). Since 2017, multiple CAR-T cells have been approved by the US Food and Drug Administration (FDA) due to their clinical success (3–6, 8, 9). However, major limitations to CAR-T cell therapy remain including severe, life-threatening CAR-T cell associated toxicities. One of these toxicities is Immune Effector Cell Associated Neurotoxicity Syndrome (ICANS). This neurotoxicity commonly occurs with CAR-T cell therapy and is the cause of significant morbidity, mortality, and cost associated with the therapy. In patients treated with CAR-T cell therapy, 20-70% develop ICANSs (3–5, 10–14). This is a phenomenon relatively unique to CAR-T cell therapy compared to regular effector T cell neurologic immune-related adverse events seen with checkpoint inhibitor therapies where approximately 4-12% of patients on checkpoint inhibitors experience neurologic immune-related adverse events and less than 1% of patients develop severe neurologic immune-related adverse events (15). Interestingly, there is no preliminary evidence of neurotoxicity or CRS with CAR-NK cell therapy, possibly suggesting a T cell unique pathophysiology (16). Here we will discuss ICANS, its treatment, possible mechanisms, and potential solutions to this critical limitation of CAR-T cell therapy. As the field of CAR-T cell therapy evolves, improved treatments and methods to circumvent or overcome ICANS are necessary to improve morbidity, mortality, and decrease the cost of CAR-T cell therapy (2, 17).

## CAR-T cell associated toxicities

While CAR-T cell therapies have had impressive success, life threatening toxicities have prevented them from becoming a first line therapy. CAR-T cells have caused significant morbidity and mortality *via* these three main toxicity syndromes (8, 9, 18, 19). The three main types of toxicities include: (a) cytokine release syndrome (CRS), which is caused by massive cytokine levels and T cell expansion; (b) hemophagocytic lymphohistiocytosis and/or macrophage activation syndrome (HLH/MAS), which is characterized as a hyperinflammatory state of CRS plus elevated serum ferritin and hemophagocytosis, renal failure, elevated liver enzymes, splenomegaly, pulmonary edema, and/or absence of NK cell activity; and (c) immune effector cell-associated neurotoxicity syndrome (ICANS), which is associated with increased cerebrospinal fluid cytokine levels and disruption of the blood-brain barrier (20). In patients treated with CAR-T cell therapy 20-70% develop ICANSs (3–5, 10–14). All FDA approved CAR-T cell products have had incidents of ICANS, thus ICANS is not a phenomena specific to anti-CD19 antigenicity (3–6). Interestingly, CD22 targeting CAR-T cells have not been associated with worse ICANS even though CD22 is expressed by human microglia (21–23).

## Grading and treatment of ICANS

Patients at greater risk for ICANs include those with younger age, pre-existing neurological/medical conditions, high tumor burden, high intensity lymphodepleting therapy, cytopenias, and early/severe CRS (14, 20, 24–28). Clinically, ICANS can present from confusion, headache, attention deficits, word finding difficulties, focal neurological deficits, or encephalopathy to life threatening cerebral edema, transient coma, or seizures (29). Classically, ICANS develops in 3 to 10 days following CAR-T cells being given (14, 20, 27). Frequently, ICANS occurs 2 to 4 days after onset of CRS, although ICANS is not required to occur in the context of CRS (14, 20, 27). Symptoms typically begin as inattention and language deficits and deterioration can rapidly progress over the course of hours to days. Usually, symptoms resolve within 7 to 10 days with appropriate treatment, but some patients will require prolonged ICU stays, and fatalities have been attributed to significant cerebral edema (14, 24, 30–33). Patients exhibit increased lactate dehydrogenase, thrombocytopenia, increased inflammatory markers, and increased cytokine levels (14, 25). Electroencephalography is often abnormal with frontal or diffuse theta-delta slowing being the most commonly observed pattern (34). In severe ICANS with increased ICP, white matter changes and sulcal effacement due to diffuse cerebral edema can be observed on imaging studies (30, 35, 36).

The American Society for Transplantation and Cellular Therapy (ASTCT) has worked to integrate various grading scales of ICANS (29, 33, 37). Grade 1 patients (mild) exhibit inattentiveness, mild disorientation, as well as mild expressive and/or receptive language dysfunction (patients can still communicate). Grade 2 patients (moderate) have moderately impaired levels of consciousness but respond to voice. Grade 3/4 patients (severe) have significant language dysfunction, respond only to tactile or noxious stimulation, and may have seizures. While the diagnose of ICANS can consistently be diagnosed using a variety of grading systems (91% of the time in one grading study), there is significant variable in grade of ICANS based on the grading scale used with only 54% of ICANS patients remaining in the same grade when using different grading scales, thus, consistent use of grading scales is critical for future trials (38). Severe ICANS (grade 3 or above) ranges from approximately 10-28% in pivotal trials in the most classically studied LBCL anti-CD19 CAR-T cells studies (39). Treatment for ICANS remains challenging, focusing mainly on supportive care and close monitoring. Steroids are the mainstay of management and are typically started with grades 2 or greater ICANS. Unfortunately, the optimal timing, dose, and duration of corticosteroids remains unclear (14, 40, 41). Furthermore, there are no targeted or prophylactic therapies to prevent any CAR-T associated toxicities, in part due to limited knowledge of ICANS pathophysiology. IL-6 blockade with tocilizumab, while an excellent treatment for CRS, has demonstrated limited utility in ICANS to date (3, 8, 9, 21, 22). Alternative cytokine-directed therapies remain under active investigation.

## Mechanisms of ICANS and possible solutions

The mechanisms behind ICANS are not well understood. Patients with severe ICANS can develop endothelial activation, disseminated intravascular coagulation, capillary leak, and blood-brain barrier permeability including increased protein and T cells in the CSF with this permeability leaving the CSF open to cytokine infiltration (23, 42). Astrocyte injury has been observed through elevated S100b and glial fibrillary acidic protein in the CSF (43, 44). The CSF has shown increased white blood cell counts, proteins, IFNγ, IL-6, IL-10, and granzyme B with serum increases in IFNγ, IL-10, granzyme B, GM-CSF, MIP-1α, and TNF (43, 44). High grade ICANS has been associated with serum elevations of GM-CSF, IL-2, and ferritin, with GM-CSF being the most associated with high grade ICANS (3, 44). There has also been a significant increase of CD14+ cells observed in patients with high grade ICANS (44, 45). Thus, it appears that a compilation of cytokines, myeloid cells, T cells, and disruption of the blood brain barrier play a role in ICANS (2, 44). A summary of ICANS markers in human patients is provided in **Table 1**.

**Table 1:** Markers of ICANS in Human Patients.

Type of Marker	Markers
Serum Markers	Lactate dehydrogenase Thrombocytopenia Ferritin IFNγ IL-10 Granzyme B GM-CSF MIP-1α TNF IL-2
CSF/Blood Brain Barrier Disruption Markers	White blood cells T cells CD14+ cells Astrocyte injury (S100b and glial fibrillary acidic protein) Increased protein permeability IFNγ IL-6 IL-10 Granzyme B
Circulatory Changes	Endothelial activation markers DIC markers
EEG Changes	Frontal or diffuse theta-delta slowing
Imaging Changes	White matter changes Sulcal effacement

One of the difficulties of studying ICANS is the limited number of animal models. In a rhesus macaque model of ICANS using CD-20-specific CAR-T cells, CRS and neurotoxicity were induced in 7-8 days (44, 46). IL6, IL8, IL1RA, MIG, and I-TAC were increased in the serum, and IL6, IL2, GM-CSF, and VEGF were disproportionately increased in the CSF (44, 46). T cells and CAR-T cells were found in the brain (44, 46). In a humanized ALL-CM leukemic cell line mouse model, mice developed a CRS like illness at around one week that lasted 2-3 weeks and increased IL-6 (44, 47). In mice that developed the CRS like illness, regardless of treatment status with the IL-6 receptor blocker tocilizumab or vehicle control, a lethal neurological syndrome developed at 30 days (44, 47). Interestingly, those mice treated with the IL-1R blocker anakinra did not develop this neurological syndrome (44, 47). The neurological syndrome was characterized by meningeal thickening without CNS infiltration of leukemic cells but infiltration of macrophages into the subarachnoid space (44, 47). In another model, a primary patient derived xenograft model of ALL was used to generate a neurotoxicity model that showed upregulation of genes responsible for controlling the T-cell receptor, cytokine receptors, T-cell immune activation, T-cell trafficking, and T-cell and myeloid cell differentiation (44, 48). Five days after anti-CD19 CAR-T cell treatment, mice exhibited T1 enhancement on MRI, which is a marker of increased blood-brain barrier permeability as well as possible edema (44, 48). These mice also exhibited neurological symptoms (44, 48). Interestingly, mice treated with GM-CSF neutralizing antibodies exhibited reduced blood-brain barrier permeability, similar to controls that did not receive anti-CD19 CAR-T cells (44, 48). T cells and macrophages were observed in the brains of mice with neurotoxicity and a decrease in raw averages was observed in mice treated with GM-CSF neutralizing antibodies (although it did not reach statistical significance) (44, 48). Thus, the timing, MRI findings, symptoms, cytokine profile, and cellular infiltration was similar in this model to what is observed in humans, and the study showed GM-CSF with neutralizing antibodies reduced neurotoxicity (44, 48). Furthermore, a CRISPR-Cas9 knockout of GM-CSF CAR-T cell was developed in these studies, which may also reduce neurotoxicity (44, 48, 49). Thus, selective control of specific cytokines may aid in the control of ICANS. In an immunocompetent mouse model of anti-CD19 CAR-T cell associated neurotoxity using escalating doses of anti-murine CD19 directed CAR-T cells (mice were pre-treated with cyclophosphamide), mice developed CRS and abnormal neurological exams 3-5 days after infusion with CAR-T cells (50). Histology showed brain hemorrhage, diffuse extravascular immunoglobulin deposition, loss of capillary pericyte coverage, and increased prevalence of string capillaries (50). *Via in vivo* two-photon imaging 6 days post CAR-T cell infusion, cortical capillary plugging and patchy hypoxia were observed in mice treated with CAR-T cells compared to mock transduced T cell treated controls (50). The capillary plugs contained CD45+ leukocytes, some of which were CD3+ T cells (50). Increased levels of soluble ICAM-1 and VCM-1 are consistent with a possible mechanism of increased leukocyte adhesion (50). This suggests a role for brain capillary obstruction impairing microvascular circulation to contribute to neurotoxicity (50).

## CAR structure and manipulation to reduce toxicities

CARs are synthetically engineered receptors that are composed of an antigen-binding domain, hinge region, transmembrane domain, and at least one intracellular signaling domain. For CAR-T cells to work effectively, the antigen binding domain must engage with the target antigen to achieve activation and cytokine production but without reaching toxic levels. The antigen expressed on malignant cells, tumor burden, antigen binding domain’s affinity to its target epitope, and the CAR’s costimulatory elements all contribute to the level of CAR activation and risk of toxicity (51, 52). These elements can be modulated through altering CAR structure in order to tune activation level and minimize the risk of toxicity.

The antigen binding domain is the portion of the CAR that confers target antigen specificity. Classically, the antigen binding domains are made from the variable heavy (V_H_) and light (V_L_) chains of monoclonal antibodies, and are connected with a linker to produce the single-chain variable fragment (scFv), which typically target extracellular surface cancer antigens resulting in major histocompatibility complex (MHC)-independent T cell activation (42, 53). The scFV also determines the affinity and specificity of the CAR for its target epitope, which ideally should be high enough to recognize antigens on tumor cells, induce CAR signaling, and activate T cells but not so high as to result in activation induced death of the CAR-T cell or trigger toxicities (54–56). Theoretically, reducing the affinity of the antigen binding domain will require the higher antigen levels in order for activation to occur. Thus, a requirement for higher levels of antigen could help reduce the targeting of healthy tissue and lessen undesired activation. Antigen binding domains with micromolar affinity have been shown to be more selective for tumors with higher antigen levels compared to those with low nanomolar/sub-nanomolar affinity (55). Another contributing factor to toxicity includes the immunogenicity of the of the antibody fragments, which can be reduced by using human or humanized antibody fragments instead of murine fragments (57).

The hinge or spacer region is the extracellular structural region that extends the binding units from the transmembrane domain. The hinge region allows flexibility to overcome steric hindrance and provides length to let the antigen-binding domain access the targeted epitope. The hinge region can affect flexibility, CAR expression, signaling, epitope recognition, strength of activation outputs, epitope recognition, and synapse formation (58–60).

The transmembrane domain anchors the CAR to the cell membrane. Importantly, the transmembrane domain may also be relevant for CAR-T cell functions such as CAR expression level, stability, dimerization with endogenous signaling molecules, and appears to play an active role in signaling or synapse formation (61–63). CAR transmembrane domains are typically derived from CD3*ζ*, CD4, CD8α, or CD28. Together, the impact of the transmembrane domain and the hinge region also appear to influence CAR-T cell cytokine production and activation induced cell death (AICD) (64). In addition, the hinge and transmembrane domains can play a role in modulating cytokine secretion. Modifying the CD8-α derived hinge and transmembrane sequences of an anti-CD19 CAR resulted in reduced proliferation and cytokine release (65). In a phase I study, 6/11 patients (54.5%) had complete remission of their B cell lymphoma and there were no CRS or ICANS events greater than grade 1 (65). The hinge and transmembrane domains may also confer immunogenicity that can contribute to toxicity (57, 66). Modification of the CAR hinge and transmembrane domains has been shown to reduce immunogenicity and enhance persistence (57, 66).

Much attention has been paid to CAR co-stimulation with the goal of generating CAR constructs with the optimal endodomain. First generation CARs contained a CD3*ζ* or FcRγ signaling domain, but this first generation did not generate robust durability and persistence (67–70). IL-2 production and proliferation was improved by adding a co-stimulatory domain (71). In second generation CARs, one co-stimulatory domain is placed in series with the CD3*ζ* intracellular signaling domain (71, 72). Among co-stimulatory domains used in second generation CARs, CD28 and 4-1BB (CD137) are the two most common and both are associated with high patient response rates (71, 72). The co-stimulatory domains differ in their functional and metabolic profiles. CARS with CD28 domains differentiate into effector memory T cells and mainly use aerobic glycolysis. CARs with the 4-1BB domain differentiate into central memory T cells and have increased mitochondrial biogenesis and oxidative metabolism (73). The 4-1BB co-stimulatory domain has been associated with lower toxicities, longer T cell endurance, and lower T cell expansion. On the other hand, the CD28 domain are linked with more rapid onset and exhaustion. Thus, the 4-1BB domain may produce less toxicity in cases of high antigen burden whereas the CD28 domain may be needed to achieve activation in low antigen density situations (74). Clinically, second generation CAR-T cells have produced strong therapeutic responses in several hematological malignancies (52). It has been hypothesized that co-stimulation through only one domain produces incomplete activation, resulting in the production of third generation CARs, which incorporate two costimulatory domains in series with CD3*ζ* (75). Preclinical studies of third generation CARs have produced mixed results (76–78).

**Figure 1:**
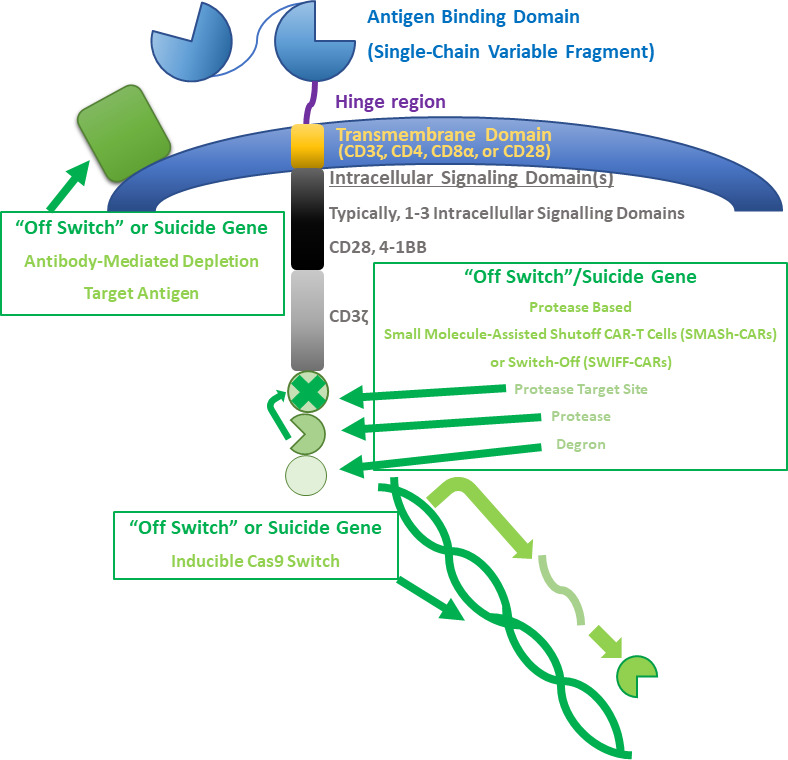
CAR-T cell structure and alterations. A summary of basic CAR-T cell structure and possible edits to alter efficacy is provided. Basic CAR-T cell structure includes an antigen binding domain, hinge region, transmembrane domain, and at least one intracellular signaling domain. These basic structures can be manipulated to alter the behavior of CAR-T cells. A few types of “off switches” or suicide genes are described in the figure. A target antigen can be expressed to allow for antibody-mediated depletion of CAR-T cells. Small molecule-assisted shutoff CAR-T cells (SMASH-CARs) or switch-Off (SWIFF-CARs) can be used to control whether CAR-T cell receptors are degraded or are able to be expressed. Cas-9 can also be inducibly expressed in some CAR-T cells.

“Off switches” or suicide genes are another strategy to stop toxicity. A secondary inducing agent can be used to selectively inhibit CAR-T cells during toxicity (79). An example of this strategy is engineering CARs to express CD20 to allow for their depletion with rituximab (80–82). A similar strategy is suggested for Cetuximab therapy for a non-signaling truncated version of EGFR to deplete CAR-T cells (83). Antibody-mediated depletion can be slow in patients who need immediate intervention during toxicities, which potentially limits this strategy. In light of this, inducible cas9 switches have been attempted, which have been shown to successfully eliminate greater than 90% of CAR-T cells in 30 minutes (84). Protease based small molecule-assisted shutoff CAR-T cells (SMASh-CARs) or switch-off (SWIFF-CARs) have also been developed (85). The main problem with these strategies, however, are that they quickly halt therapy, which could be a problem in rapidly progressing disease. Thus, suicide gene engagement could serve as a last resort for safety. A summary of basic CAR-T cell structure and possible edits to alter efficacy is provided in **Figure 1**.

## Discussion

Immune Effector Cell Associated Neurotoxicity Syndrome (ICANS) is a potentially life-threatening neurotoxicity that commonly occurs with CAR-T cell therapy. Clinically, ICANS can present with clinical symptoms ranging from confusion, headache, attention deficits, word finding difficulties, focal neurological deficits or encephalopathy, to life threatening cerebral edema, transient coma, or seizures (29). Supportive care and corticosteroids are the mainstays of treatment, but the optimal timing, dose, and duration of corticosteroids has not been determined (14, 40, 41). Although the precise mechanisms of ICANS are not known, disruption of the blood-brain barrier, cytokines, myeloid cells, and T cells have all been suggested to play a role. In the future, blocking some of these cytokines networks or designing CAR-T cells structurally to be less toxic are strategies to potentially one day overcome ICANS. In this review we discussed ICANS, its treatment, possible mechanisms, and potential solutions to this critical limitation of CAR-T cell therapy. As the field of CAR-T cell therapy evolves, improved treatments and methods to circumvent or overcome ICANS are necessary to improve morbidity, mortality, and decrease the cost of CAR-T cell therapy. This serious, life-threatening side effect requires further investigation in the future in order to better understand its mechanisms and develop treatments and alternative strategies.

## Author contributions

RCS and RMS designed, wrote, edited, and approved the final version of the manuscript. RMS supervised the study. All authors contributed to the article and approved the submitted version.

## Funding

This work was supported through Regenerative Medicine Minnesota RMM 012819 EPC 003 (RMS). RCS was supported by 1 F30 MH124284-01.

## Conflict of interest

RMS is an inventor on patents related to CAR-T cell therapy licensed to Humanigen through Mayo Clinic.

The remaining author declares that the research was conducted in the absence of any commercial or financial relationships that could be construed as a potential conflict of interest.

## Publisher’s note

All claims expressed in this article are solely those of the authors and do not necessarily represent those of their affiliated organizations, or those of the publisher, the editors and the reviewers. Any product that may be evaluated in this article, or claim that may be made by its manufacturer, is not guaranteed or endorsed by the publisher.
